# Convolutional Neural Networks Promising in Lung Cancer T-Parameter Assessment on Baseline FDG-PET/CT

**DOI:** 10.1155/2018/1382309

**Published:** 2018-10-30

**Authors:** Margarita Kirienko, Martina Sollini, Giorgia Silvestri, Serena Mognetti, Emanuele Voulaz, Lidija Antunovic, Alexia Rossi, Luca Antiga, Arturo Chiti

**Affiliations:** ^1^Department of Biomedical Sciences, Humanitas University, Milan, Pieve Emanuele, Italy; ^2^Nuclear Medicine, Humanitas Clinical and Research Center, Milan, Rozzano, Italy; ^3^Orobix Srl, Bergamo, Italy; ^4^Thoracic Surgery, Humanitas Clinical and Research Center, Milan, Rozzano, Italy; ^5^Radiology, Humanitas Clinical and Research Center, Milan, Rozzano, Italy

## Abstract

**Aim:**

To develop an algorithm, based on convolutional neural network (CNN), for the classification of lung cancer lesions as T1-T2 or T3-T4 on staging fluorodeoxyglucose positron emission tomography (FDG-PET)/CT images.

**Methods:**

We retrospectively selected a cohort of 472 patients (divided in the training, validation, and test sets) submitted to staging FDG-PET/CT within 60 days before biopsy or surgery. TNM system seventh edition was used as reference. Postprocessing was performed to generate an adequate dataset. The input of CNNs was a bounding box on both PET and CT images, cropped around the lesion centre. The results were classified as Correct (concordance between reference and prediction) and Incorrect (discordance between reference and prediction). Accuracy (Correct/[Correct + Incorrect]), recall (Correctly predicted T3-T4/[all T3-T4]), and specificity (Correctly predicted T1-T2/[all T1-T2]), as commonly defined in deep learning models, were used to evaluate CNN performance. The area under the curve (AUC) was calculated for the final model.

**Results:**

The algorithm, composed of two networks (a “feature extractor” and a “classifier”), developed and tested achieved an accuracy, recall, specificity, and AUC of 87%, 69%, 69%, and 0.83; 86%, 77%, 70%, and 0.73; and 90%, 47%, 67%, and 0.68 in the training, validation, and test sets, respectively.

**Conclusion:**

We obtained proof of concept that CNNs can be used as a tool to assist in the staging of patients affected by lung cancer.

## 1. Introduction

In recent years, advanced analysis of medical imaging using radiomics, machine, and deep-learning, including convolutional neural networks (CNNs), has been explored. These approaches offer great promise for future applications for both diagnostic and predictive purposes. CNNs are nonexplicitly programmed algorithms that identify relevant features on the images that allow them to classify an input object. They have been applied in various tasks such as detection (e.g., breast lesions on mammographic scans), segmentation (e.g., liver and liver lesions on computed tomography (CT)), and diagnosis (e.g., lung lesions on screening low-dose CT).

CNNs are a machine-learning technique based on an artificial neural network with deep architecture relying on convolution operations (the linear application of a filter or kernel to local neighbourhoods of pixel/voxels in an input image) and downsampling or pooling operations (grouping of feature map signals into a lower-resolution feature map). The final classification or regression task relies on higher-level features representative of a large receptive field that is flattened into a single vector. The development of an algorithm entails (a) selection of the hyperparameters, (b) training and validation, and (c) testing. The *hyperparameters* include the network topology, the number of filters per layer, and the optimisation parameters. During the *training* process, the dataset of input images (divided into training and validation sets) is repeatedly submitted to the network to capture the structure of the images that is salient for the task. Initially, the weights for each artificial neuron are randomly chosen. Then, they are adjusted at each iteration, targeting minimisation of the loss function, which quantifies how close the prediction is to the target class. The performance of the trained model is then evaluated using an independent *test* dataset. This is also aimed at assessing whether an “overfitting” has occurred. The overfitting problem can arise in the case of limited datasets with too many parameters compared with the dataset size, in which case a model “memorises” the training data rather than generalising from them [1].

In the field of lung imaging, CNNs have been tested in nodule segmentation from CT images. Average dice scores of 82% and 80% for the training and test datasets, respectively, have been reported [2]. CNNs have been demonstrated to achieve better results than conventional methods for the purpose of nodule detection [3, 4]. Moreover, a model for assessment of cancer probability in patients with pulmonary nodules has been proposed. The area under the curve (AUC) was found to be 0.90 and 0.87 for the training and test sets, respectively [5]. Stage assessment has not yet been described. The present study, as a first step towards complete TNM parameter assessment, aimed to develop an algorithm for the classification of lung cancer as T1-T2 or T3-T4 on staging fluorodeoxyglucose positron emission tomography (FDG-PET)/CT images.

## 2. Materials and Methods

### 2.1. Study Design and Patient Selection

In this retrospective single-centre investigation, we screened all patients who underwent FDG-PET/CT between 01/01/2011 and 27/06/2017 for the purpose of staging a suspected lung lesion, within 60 days before biopsy or surgical procedure. The inclusion criteria were (1) age > 18 years and (2) histological diagnosis of primary lung cancer. The exclusion criteria were (1) inconclusive histology due to inadequate biopsy sample and (2) diagnosis of nonmalignancy. The study was approved by the Institutional Ethics Committee.

### 2.2. Image Acquisition and Postprocessing

FDG-PET/CT was performed according to the standard institutional procedures, previously detailed [6]. Postacquisition processing was performed to generate an adequate dataset for the CNN. The original CT and PET image size was 512 × 512 × *N*_slices_ and 128 × 128 × *N*_slices,_ respectively, where *N*_slices_ is the number of slices in which the lesion appears. The CT images were clipped between −1000 and 400 Hounsfield units. PET images were resampled in the CT space. Then, both images were rescaled to lie between 0 and 1. Consequently, the dataset consisted of 3D bounding boxes on both PET and CT images, cropped around the lesion centre, identified by two nuclear medicine physicians (M.S. and M.K.) with dimension 128 × 128 × *N*_slices_. Data augmentation, a strategy commonly used by deep-learning methods, was performed. Image patches were rotated in 2D space around the lesion centre about the *z*-axis by an angle randomly selected in a range of [−10°, 10°]. This processing step artificially expands the size of the training set and reduces the overfitting phenomena.

### 2.3. CNN Development and Analysis

**Figure 1:**
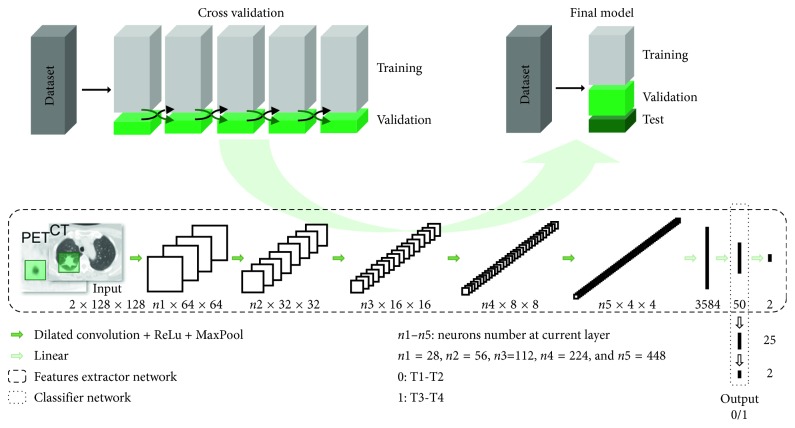
Study workflow and networks' architecture. The network (consisting of two neural networks, the feature extractor, and the classifier) was trained using a cross-validation strategy. The entire dataset was divided into 5 randomly chosen parts. At each training run, 4/5 of the dataset were used as a training set and the remaining 1/5 was used as the validation set. Subsequently, the final model network was adjusted and tested for performance using the dataset divided into three sets: training, validation, and test. In the *feature extractor* CNN, the input images, both PET and CT, were submitted to a series of convolutions producing a stack of feature maps containing low-level features, rectified linear units (ReLU), and max pooling layers that downsample the feature maps (MaxPool), to produce higher-level features. In the *classifier* network, these higher-level features are used to perform the final classification T1-T2 (output label 0) vs T3-T4 (output label 1).

The study workflow and the networks' architecture are summarised in Figure 1. During the training phase, a fivefold cross-validation strategy was adopted by dividing the cohort into a training dataset and a validation dataset. To assess the performance of the final model, the cohort was divided into training, validation, and test datasets. The algorithm was composed of two networks: a *feature extractor* and a *classifier*. The *feature extractor* was a CNN that took a CT-PET image patch of 128 × 128 pixels as input and performed classification (T1-T2 with label = 0 and T3-T4 with label = 1) according to the appearance of the image patch. The *feature extractor* aimed to extract the most relevant features from a single patch. The *classifier* took as input the mean of the second to last layer of features extracted from all slices of a single patient and aimed to perform a classification (T1-T2 vs. T3-T4) for that patient. The *softmax function* was applied to the last layer of both networks, in order to obtain the probability of being T1-T2 and T3-T4. The class having the highest probability was assigned to each patient. Both models were trained with the Adam algorithm [7].  Table 1 summarises the parameters for the *feature extractor* and the *classifier* networks. The code was written in Python, using the PyTorch deep-learning library (http://pytorch.org/).

TNM classification system 7^th^ edition [8] was used as reference.

The results were classified as Correct (concordance between reference and prediction) and Incorrect (discordance between reference and prediction). Accuracy (Correct/[Correct + Incorrect]), recall (Correctly predicted T3-T4/[All T3-T4]), and specificity (Correctly predicted T1-T2/[all T1-T2]) as commonly defined in deep-learning models, were used to evaluate CNN performance. The area under the curve (AUC) was calculated for the final model.

## 3. Results

From the institutional database, a cohort of 586 patients was selected by applying the abovementioned criteria. Patients with distant metastases or histology different from adenocarcinoma and squamous cell carcinoma were excluded from the present analysis. Therefore, 472 patients were included in the study (T1-T2 = 353 patients, T3-T4 = 119 patients). Staging was clinical and pathological in 97 and 375 of cases, respectively. Subsequently, the patients were randomly divided in training (*n*=303), validation (*n*=75), and test (*n*=94) sets. The patients' characteristics are summarised in Table 2.

Table 3 summarises the results of the cross-validation analysis.

The algorithm developed and tested in the present work achieved an accuracy of 69%, a recall of 70%, and a specificity of 67% in the test set, for the identification of T1-T2 and T3-T4 lung cancer, in the final model analysis. The AUC was 0.83, 0.73, and 0.68 in the training, validation, and test sets, respectively. Results of all metrics for the final model in the training, validation, and test sets are reported in Table 4.

**Figure 2:**
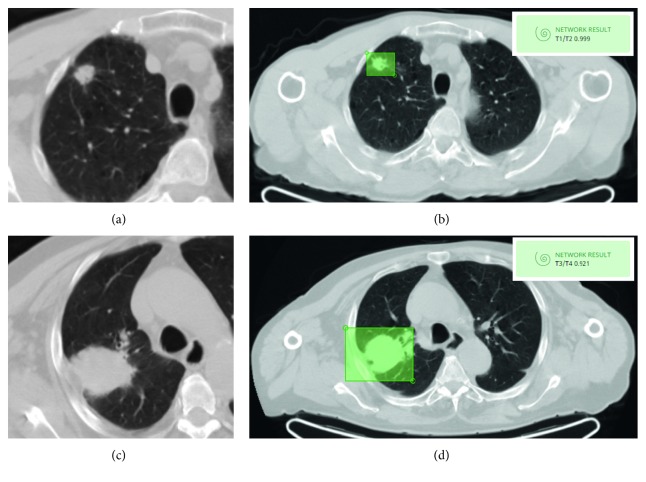
Clinical examples of the network classification. Axial CT images (a, b) of a patient affected by lung adenocarcinoma pT1bN0 ((a) zoom on the lesion); the developed neural network classified the new input image ((b) green square on one of the slices in which the lesion is visible) as belonging to the class T1-T2 with 99% probability. Axial CT images (c, d) of a patient affected by lung adenocarcinoma pT4N1 ((c) zoom on the lesion); the developed neural network classified the new input image ((d) green square on one of the slices in which the lesion is visible) as belonging to the class T3-T4 with 92% probability.

Figure 2 shows examples of patients classified by CNN as T1-T2 and T3-T4.

## 4. Discussion

The algorithm developed and tested in the present work achieved an accuracy of 87%, 69%, and 69% in the training, validation, and test sets, respectively, for the classification of T1-T2 and T3-T4 lung cancer. The lower performances of the CNN in the validation and test sets compared with the training dataset are probably related to the sample size (*n*=75, *n*=94, and *n*=303, respectively).

The TNM staging system is the reference method for prognostication and treatment decision-making in cancer patients, including in those with lung cancer. Pathological assessment is considered the gold standard. However, patients with tumours of the same stage can experience variations in the incidence of recurrence and survival. These variations may be related to tumour biology and other factors, including potential differences in the pathological stage at diagnosis and at neoadjuvant treatment [9] and the possible effect of the number of surgically removed lymph nodes on the N-parameter [10]. Finally, pathological TNM staging is not feasible in advanced stages. Medical imaging is the reference when pathological assessment is not feasible. Hybrid FDG-PET/CT is noninvasive and provides whole-body assessment, resulting essential in baseline lung cancer staging. CT scan is the cornerstone of lung cancer imaging providing all the necessary information needed for clinical T staging. FDG-PET/CT outperforms other modalities in terms of diagnostic accuracy in mediastinal nodal involvement and extrathoracic disease detection [11, 12].

In lung CT imaging, deep-learning approaches have been used to detect lung nodules [3, 13], to segment [2], and to classify them as benign or malignant [5, 14]. Some preliminary data are available on radiation treatment planning [15] and outcome prediction [16]. Recently, the value of CNNs for classification of mediastinal lymph node metastasis on FDG-PET/CT images has been investigated in non-small cell lung cancer. CNNs proved promising in identifying lymph node involvement, with higher sensitivities but lower specificities compared with doctors [17].

The present work reports an innovative application of deep-learning providing an automated staging classification. Some limitations of this study have to be acknowledged. Firstly, the limited number of patients precluded the design of a network for a classification task with four outputs (T1, T2, T3, and T4). Moreover, better performance is expected in larger datasets. In future work, we plan to include a larger number of cases in a multicentre framework. Secondly, the reference to define the T-parameter was based on the pathological assessment in surgically treated patients (80% of cases), while in patients not suitable for surgery, clinical assessment was used as the standard. The choice to use the pathological staging when available instead of the clinical one was aimed to achieve higher algorithm performance. Finally, we did not test separately CT and PET image modality. It could be speculated that, in this first step investigation, the CNN nodes were mostly activated by the weighted features coming from the CT component, while for the N- and M-parameters, PET can be supposed to give a major contribution. The comparison between the performances of a CNN trained using either CT or PET images will be considered in future studies. However, our final objective is to develop a whole-body assessment tool processing both CT and PET images together.

In conclusion, the key result in the present preliminary investigation is the feasibility and promising performance of CNNs in assessing the T-parameter in lung cancer. The developed tool is able to provide in few minutes from baseline PET/CT the probability of a patient being T1-T2 or T3-T4. Further investigations are needed to develop robust algorithms for a complete TNM assessment. Compared with radiomics, CNNs have the advantage of eliminating the need for tumour segmentation, feature calculation and selection, which are even more critical issues in small lesions. Still, the possibility of a complementary role of radiomics and artificial intelligence techniques should be addressed. Moreover, improvement in risk stratification is foreseen with the incorporation of patients' clinical features in the neural network algorithm.

## Figures and Tables

**Table 1:** Summary of the parameters of the feature extractor and the classifier networks.

	Feature extractor	Classifier
Loss function	Cross entropy	Weight cross entropy
Learning method	Adam	Adam
Learning rate	1*e* − 6	1*e* − 3
Weight decay	1*e* − 6	1*e* − 6
Epochs	600	600
Patients/batch	2	2
Slides/patient	20	n.a.

n.a.: not applicable.

**Table 2:** Patients' characteristics.

Characteristics	Patients, *n* (%)
Age (year): mean: 69 ± 9, median: 69 (range: 36–112)	

*Sex*	
Male	316 (67)
Female	156 (33)

*Histology*	
Adenocarcinoma	317 (67)
Squamous cell carcinoma	155 (33)

*TNM*	
T1	159 (34)
T2	194 (41)
T3	79 (17)
T4	40 (8)
N0	259 (55)
N1	94 (20)
N2	103 (22)
N3	16 (3)
M0	472 (100)

*Stage* ^*∗*^	
I	193 (41)
** **Ia	115 (24)
** **Ib	78 (17)

II	116 (25)
** **IIa	66 (14)
** **IIb	50 (11)

III	163 (34)
** **IIIa	129 (27)
** **IIIb	34 (7)

^*∗*^The stage was clinically assessed in 97 patients (20%), while in the remaining 375 cases (80%), it was pathologically assessed.

**Table 3:** Results of the cross-validation analysis.

	Training (*n* =378)			Validation (*n* =94)		
	Accuracy (%)	Recall (%)	Specificity (%)	Accuracy (%)	Recall (%)	Specificity (%)
CV 1	82.6	91.2	56.8	76.6	84.3	54.2
CV 2	87.3	95.8	62.1	76.6	91.4	33.3
CV 3	78.4	91.2	40.6	81.9	90.0	58.3
CV 4	85.0	88.3	75.0	73.4	81.7	47.8
CV 5	79.7	94.0	36.8	78.7	94.3	33.3
Mean	82.6	92.1	53.4	77.4	88.3	45.4

CV: cross validation.

**Table 4:** Results of the final model.

	Accuracy (%)	Recall (%)	Specificity (%)	Area under the curve
Training (*n* =303)	86.8	85.9	89.5	0.83
Validation (*n* =75)	69.3	76.8	47.4	0.73
Test (*n* =74)	69.1	70.0	66.7	0.68

## Data Availability

The data used to support the findings of this study are available from the corresponding author upon request.

## Additional Points

*Clinical Relevance*. The development of an automated system based on neural networks, that is, the ability to perform a complete stage and risk assessment will ensure more accurate and reproducible use of imaging data and allow deeper integration of this information in the therapeutic plan for each individual patient.
